# Associations Between Psychological Coping Skills and Player Behaviors During Transition Moments in Male Youth Football

**DOI:** 10.3390/sports13100363

**Published:** 2025-10-13

**Authors:** Francisco Pires, Maria Inês Vigário, Sandra S. Ferreira, António Vicente

**Affiliations:** 1 Department of Sports Sciences, University of Beira Interior, 6200-001 Covilhã, Portugal; 2 Research Center in Sports Sciences, Health Sciences and Human Development (CIDESD), 5001-801 Vila Real, Portugal; 3 Portuguese Olympic Committee, 1300-403 Lisboa, Portugal; 4 Department of Mathematics, Centre of Mathematics, University of Beira Interior, 6200-001 Covilhã, Portugal

**Keywords:** coping skills, diagnose, football, match analysis, psychological resilience, sports-based interventions, youth mental health, youth sport psychology

## Abstract

Sport performance results from the interaction of tactical, technical, physiological and psychological factors, but psychological aspects are often minimized or analyzed in a decontextualized manner. This exploratory pilot study aimed to contribute to the development of a diagnostic framework that links individual behaviors during football attack–defense transition moments (ADT) with psychological attributes. Twenty male U14 players were assessed across five official matches regarding their ADT performance indicators. The Athletic Coping Skills Inventory (ACSI-28) and the Resilience Scale (RS) were applied during the competition. Statistical analyses included correlation tests and Bayesian analysis. Players showed a significant tendency to sustain ball recovery behaviors after possession loss (*p* = 0.004). Psychological resilience and athletic coping skills varied substantially between individuals without positional differences, as well as RS scores were significantly below the high-resilience threshold (147; *p* = 0.013). A moderate positive correlation emerged between RS Factor 1 and the ACSI-28 subscale “Coping with Adversity” (r = 0.574, *p* = 0.008). Posterior distributions provide exploratory signals suggesting possible positive associations for two psychological constructs considering ADT individual behaviors: “Concentration” in relation to the maintenance of recovery actions (Mode = 0.439; 95% CI [0.030, 0.721]) and “Goal Setting” in relation to the rapid initiation of recovery actions (Mode = 0.465; 95% CI [0.059, 0.734]). Nevertheless, Bayes Factors favored the null model overall, indicating that these signals are weak and require replication. By contrast, most psychological constructs, including resilience, showed no reliable evidence of correlation with recovery-related actions. The findings highlight the need to further research the integration of psychological assessment into football performance diagnostics, while also indicating that psychological factors alone are insufficient to fully explain youth players’ individual ADT behaviors.

## 1. Introduction

### 1.1. Background

Sport performance is a multifactorial phenomenon shaped by the dynamic interaction of technical, tactical, physiological and psychological dimensions, requiring holistic assessment and intervention [1]. While this complexity is increasingly acknowledged in scientific discourse, practical applications, particularly in youth football, often emphasize physical or tactical preparation over psychological components [2], despite growing evidence supporting its critical role in competitive contexts and successful youth development [3].

Psychological competencies/skills are critical for optimal performance, influencing performers’ ability to manage stress, adapt to challenges and sustain competitive consistency [4,5].

In football practice and match environments, these competencies/skills are particularly relevant during demanding situations, such as attack–defense transition moments (ADT), where decision-making and behavioral responses must be rapid and adaptive [6]. Assessing psychological competencies/skills like resilience and athletic coping skills within these contexts might provide insights into how young players manage pressure and recover from adversity [7].

Moreover, considering the significant role of practice in determining competition outcomes, the urgency to assess the usage of psychological competencies/skills in both practice and competition environments is real [5].

### 1.2. Literature Review and Research Gap

Psychological resilience, once conceptualized as a stable personality trait, is now regarded as a dynamic process shaped by interactions between personal and environmental factors [4,5,6,7,8,9,10]. It has been associated with positive adaptations to adversity, including enhanced confidence, motivation, focus and social support [4,5,6,7,8,9,10,11]. It is also linked to better mental health and reduced vulnerability to stress and anxiety [10,11,12].

The existing literature indicates that, when examining psychological resilience levels among youth sports practitioners across different competitive levels, those engaged in higher-level competitions tend to demonstrate greater resilience [13]. However, evaluating this psychological construct in adolescent populations raises several issues that must be addressed with great caution [14].

On the other hand, psychological skills, namely athletic coping skills, have been identified as predictors of performance and well-being in sport practitioners [7,8,9,10,11,12,13,14,15], with evidence indicating that their effective use is positively associated not only with performance outcomes but also with mental health markers, including reduced anxiety and injury risk. Nevertheless, the literature reports contradictory findings regarding potential differences in coping skills among football players, depending on their specific roles and playing positions on the field [13,14,15,16,17]. Certainly, this variable alone is insufficient to account for the differences observed in players’ psychological profiles.

Age-related differences have also been reported, particularly within the cohorts targeted in the present study: compared with U14 players, U15 players generally display more positive athletic and pain coping skills, reflecting the possible influence of accumulated experience [16].

Psychological coping skills have additionally been associated with the capacity to adapt to training demands, regulate competitive anxiety and sustain motivation [5,6,7]. These skills could be particularly relevant in football, where transition moments can represent stressful situations.

Despite these findings, most assessments of psychological competencies/skills in sport rely on indirect measures (e.g., questionnaires, interviews), with limited efforts to integrate observable behaviors from competitive contexts into psychological profiling [3,4,5,6,7,8,9,10,11,12,13,14,15,16,17,18,19].

In youth football, psychological resilience is considered a key psychosocial competence for development and success [3,4,5,6,7,8,9,10,11], as well as athletic coping skills, yet research connecting individual behavioral data from practice/match situations with psychological profiles remains scarce [19].

Understanding these relationships could inform both performance optimization and mental health promotion in young players [20,21,22].

Although some studies have attempted to relate psychological constructs to performance outcomes at both individual and collective levels (e.g., batting averages in baseball [23]), the specific associations between these constructs and observable players’ actions remain underexplored.

In summary, notwithstanding the recognized importance of psychological competencies/skills in sport, research linking them to in-match behaviors is still limited. Existing reliance on self-report measures, while informative, offers limited insight into how these resources manifest during competition.

This gap is especially relevant in youth football, where varied demands and formative experiences shape both immediate performance and long-term development. Therefore, different approaches integrating psychological assessment with observed behaviors are needed to better understand how psychological resilience and coping skills translate into action in the field.

Additionally, studying under 14-year-old players offers a timely opportunity: at this developmental stage, players are moving from different competition formats (8v8, 9v9) to formal football competition (11v11), usually passing through maturational transformations, coping with new pressures while still building fundamental psychological competencies/skills.

Understanding how psychological resilience and athletic coping skills manifest in key match moments—such as ADT—at this developmental stage can provide early diagnostic insights, informing individualized support and interventions.

### 1.3. Research Questions, Hypotheses, Aim, and Innovation

This pilot study aims to bridge the identified research gap by integrating match analysis with psychological assessment to examine the possible relationships between psychological resilience, athletic coping skills and individual behaviors of under-14 male football players during ADT. Specifically, it seeks to perform the following:

(a) Characterize players’ individual behaviors during ADT, psychological resilience and athletic coping skills profiles;
(b) Examine eventual positional differences in psychological resilience and athletic coping skills profiles;
(c) Explore associations between Athletic Coping Skills Inventory (ACSI-28) coping dimensions and Resilience Scale (RS) scores;
(d) Investigate the possible associations between psychological resilience, athletic coping skills and players’ behaviors during ADT;
(e) Identify psychological dimensions most strongly associated with behavioral success in ADT.

It was hypothesized that players with higher psychological resilience and athletic coping skills would be more likely to show associations with consistent defensive intentions and recovery-oriented behaviors during ADT, irrespective of playing position.

By combining behavioral indicators with validated psychological measures, this exploratory pilot study, interpreted as a hypothesis generator, proposes a methodological framework that reflects the complexity of sport performance, offering potential applications for both performance enhancement and youth mental health support. This approach underscores the value of interdisciplinary and ecological perspectives in advancing diagnostics and interventions in youth sport [24].

## 2. Materials and Methods

### 2.1. Study Design

This pilot study, exploratory in nature, used a correlational design to examine the relationship between two data sets: match performance indicators and psychological questionnaire results [25]. Descriptive analyses were also conducted to provide an overview of the youth players’ behavior patterns at ADT and psychological profiles.

Although the study does not allow for generalizable conclusions, it could offer useful insights into the possible associations between the examined variables and generate hypotheses to test in the future. Additionally, it contributes to evaluating the consistency of the tested methodology.

While the present sample is limited to a single team, its ecological validity derives from analyzing a real competition context. Rather than aiming for generalizability, this exploratory initiative tests a correlational design and a methodological framework that could be scaled to other teams, age groups and contexts.

By piloting the integration of psychological profiling with match analysis of individual behaviors, this study contributes to advancing both sport psychology and applied performance science, offering a foundation for future research with larger and more diverse samples.

### 2.2. Sample

The study sample comprised twenty (N = 20) male football players under 14 years of age (U14) from the same team, who participated in the 2023/2024 Lopes da Silva Interassociations Tournament from the Portuguese Football Association. The participants had a mean age of 14.0 years (SD = 0.51).

A non-probability convenience sampling method was employed.

The inclusion criteria for the final study sample were: (1) being an official player registered on the U14 team roster of the selected Regional Association; (2) having participated in at least three of the five matches recorded during the observation period; and (3) having completed the psychological questionnaires (RS and ACSI-28) in full. No formal exclusion criteria were applied, although players who did not meet the inclusion conditions were not considered in the final analysis.

All players who met these inclusion criteria were included in the study sample and no players were excluded arbitrarily. Therefore, the sampling method can be classified as a total sampling of all eligible participants within the team during the tournament period.

As a result, the final sample consisted of 20 players meeting all eligibility requirements. This sampling approach ensured that the sample accurately represented the active players who were psychologically assessed and participated in most matches during the study timeframe.

### 2.3. Tools

#### 2.3.1. Match Data Analysis Matrix

The Match Data Analysis Matrix used to evaluate players’ behaviors (Appendix A) was developed and validated through a multi-phase process including expert input, the literature review and pilot testing, ensuring its suitability for this population.

Initially, top-level football coaches were surveyed regarding the importance of evaluating practitioners’ behaviors and psychological competencies/skills in competition contexts, which guided the development of an observational instrument [26]. Following this, a comprehensive literature review facilitated the construction and development of a grid-based analysis tool tailored to football transition moments [26]. The instrument’s content validity was established through collaboration with two football experts, and its feasibility and applicability were confirmed in a pilot study covering three games of the same team [27].

To ensure consistent performance measurement, explicit operational definitions and timing criteria were adopted for coding transition actions within the observational grid. Although inter-rater reliability coefficients were not calculated, coding was conducted by two trained observers with periodic consensus meetings, as recommended in established observational research protocols [28]. This approach helps ensure the robustness and consistency of the observational data, but it should be considered a limitation to be addressed in future research.

Within the scope of this work, the focus was only on ADT data, considering that this specific moment could represent a stressor (linked to the psychological resilience process) and deducing that a consequence in players’ behavior could be visible, identified and measured. This option, necessary to enable dealing with a less comprehensive set of information that allows to draw well-founded conclusions within the available time frame, arises from the perception that many of the observable actions in these situations may be related to behaviors already identified as being associated with certain psychological competencies/skills (namely, those related to the concept of sporting psychological resilience) [11].

Hence, based on players’ visible actions—and supported by the necessary conceptual framework to understand in a contextualized way the meaning and significance of these same actions—the diagnosis of these competencies/skills can be conducted through performance complexity based on identified behavioral trends. Consequently, supported in a conjecture-refutation logic that does not exclude hypotheses and allows for establishing causes between different performance factors, this tool enhances a holistic and ecological approach to these phenomena.

#### 2.3.2. Athletic Coping Skills Inventory

The ACSI-28 [15] has the specific intention of evaluating individual differences in psychological skills within the sporting context. This instrument includes 28 items answered on a 4-point Likert scale (0 = Rarely to 3 = Almost always). The 28 items are distributed across 7 subscales (with 4 items each), and each of them assesses the following psychological skills:

(a) Maximum performance under pressure (items 6, 18, 22, 28);
(b) Absence of worries (items 7, 12, 19, 23);
(c) Confrontation with adversity (items 5, 17, 21, 24);
(d) Concentration (items 4, 11, 16, 25);
(e) Objectives’ formulation (items 1, 8, 13, 20);
(f) Confidence and motivation to achieve (items 2, 9, 14, 26);
(g) Availability to learn from training (items 3, 10, 15, 27) [15].

Specifically related to football contexts, the ACSI-28 items could be described as follows:

(a) Maximum performance under pressure: feels more challenged than threatened in situations of competitive pressure to achieve good levels of performance under pressure;
(b) Absence of worries: you do not put pressure on yourself, worrying about poor performance or mistakes you might make, nor do you worry about what others might think if you perform poorly;
(c) Dealing with adversity: remains positive, calm, controlled and enthusiastic even when things go wrong; can recover quickly from mistakes and setbacks;
(d) Concentration: not easily distracted and able to focus on tasks even when adverse or unexpected situations occur;
(e) Defining objectives and mental preparation: formulates short-term objectives and works to achieve concrete performance objectives, planning and mentally preparing for the competition;
(f) Confidence and motivation: is confident and positively motivated and gives all his effort to improve;
(g) Coachability: open and available to learn from what is taught in training, accepting criticism from coaches and managers positively and constructively [29].

The ACSI-28 also allows the extraction of a total “score” (resulting from the seven sub-scales ‘scores’ sum), which constitutes a measure of Personal Confrontation Resources in sporting competition and which reflects a multifaceted estimate of the practitioner’s skills [30].

The study of the relations between ACSI-28 and the Resilience Scale (RS), for example, may be an interesting field in sport psychology research. However, the specific combination of these two instruments is not widely documented. Both questionnaires can be used in sporting contexts to assess psychological competencies/skills that may be interrelated, anticipating that the intersection of their dimensions could offer a promising research field, given the crucial role that confrontation resources and resilience seem to have in sporting performance.

The ACSI-28 total scale yields high internal consistency, Alpha, and test–retest coefficients (above 0.80) [31].

To examine consistency within this study data set (N = 20), Cronbach’s Alpha was calculated (α = 0.816). The result indicates very acceptable internal consistency, but it is important to stress that estimates derived from such a small group are inherently unstable and must be read with caution.

#### 2.3.3. Resilience Scale

The RS is an instrument designed to assess individual resilience, conceptualized as a positive personality characteristic that facilitates adaptation [14,32,33]. It comprises 25 positively worded items rated on a 7-point Likert scale, ranging from 1 (totally disagree) to 7 (totally agree). Total scores can range from 25 to 175, with higher scores indicating greater resilience. Scores equal to or above 147 are classified as high resilience [14,15,16,17,18,19,20,21,22,23,24,25,26,27,28,29,30,31,32,33].

Subscale calculations for the RS followed the published two-factor structure validated for the Portuguese population. The Portuguese Version of the RS used in this research follows its established two-factor structure as validated by previous studies in Portuguese populations [14,32]. In those validation studies, principal component analysis with oblique (Oblimin) rotation identified two factors: ‘Personal Competence’ (17 items: 1, 2,…), and ‘Acceptance of Self and Life’ (8 items: 7, 8, …).

For the present sample (N = 20), we calculated subscale scores according to the published model, as our sample size was insufficient for conducting reliable factor analysis.

Prior studies report strong internal consistency (total scale α = 0.83; subscale α = 0.75) and construct validity [34].

Internal consistency was evaluated on the sample (N = 20) by Cronbach’s Alpha, which yielded a coefficient of α = 0.78. While this result suggests acceptable internal consistency, the limited sample size precludes robust inferential interpretations.

No new factor extraction was performed on the present data.

### 2.4. Procedures

Data from ADT of five (5) Lopes da Silva Tournament matches were collected and analyzed (through video), using a specific matrix for this purpose, in which, summarizing all these 5 matches, all the 20 players participated (although with different utilization times).

The videos were recorded using a Canon Legria HF R506 video camera by Canon Inc., Tokyo, Japan (Full HD 1080p, 50p, AVCHD and MP4), positioned at an elevated vantage point located centrally along the length of the field.

All players were also asked to answer the ACSI-28 [15] and RS [14,33] during the competition.

Before data collection, an explanatory document about the purposes of this study, as well as the consent that allowed the players’ inclusion, was distributed to their guardians.

All the procedures are carried out in due respect by the Helsinki Declaration and allowed by the Ethical Commission of Beira Interior University with the process number CE-UBI-Pj-2024-039.

### 2.5. Statistical Analysis

Given the exploratory and pilot nature of the study and the limited sample size (N = 20, with very small subgroups such as goalkeepers: N = 2), statistical analyses were planned to prioritize robustness under small-sample conditions while also maintaining comparability with established benchmarks.

Descriptive statistics (means, standard deviations, ranges) were first calculated for all variables. For inferential testing, different approaches were combined, each selected according to the type of question addressed.

Normality and homogeneity tests (Shapiro–Wilk, Levene) were conducted only for descriptive purposes, but not used as criteria to switch between parametric and non-parametric approaches, given their low reliability in small-N contexts.

Wilcoxon signed-rank tests were used for within-player comparisons (e.g., % Maintained Intention vs. % Slowed/Cancelled Intention), as they are a robust non-parametric test to violations of normality in small-N designs.

Kruskal–Wallis tests were applied for between-group comparisons (e.g., positional differences), given the small sample sizes per group and the non-normal distributions of some variables. Effect sizes (ε^2^) were reported to complement *p*-values.

Associations between psychological variables were examined using Spearman’s rank correlations, more appropriate for ordinal or non-normally distributed data.

One-sample *t*-tests were retained only for direct comparisons against an external benchmark (e.g., the RS high-resilience cutoff = 147). Although parametric, this choice allows a direct statistical test of whether the sample mean differed from the predefined threshold. As a robustness check, effect sizes (ε^2^) were emphasized and results were interpreted cautiously.

Bayesian inference was employed to complement frequentist tests, as it is particularly suitable for small samples [35,36,37]. Bayes Factors (BF10) and posterior distributions were reported to provide a continuous measure of evidence, rather than relying solely on dichotomous significance thresholds. Non-informative priors were used, and interpretation followed conventional guidelines (BF10 < 0.33 = evidence for null, 0.33–3 = inconclusive, BF10 > 3 = evidence for alternative).

This combined strategy was chosen because no single method fully addresses the challenges of small-sample, exploratory designs. Frequentist tests enable comparability with previous studies, while Bayesian inference provides additional nuance by quantifying the strength of evidence in favor of either hypothesis [35,36,37]. In all cases, emphasis was placed on effect sizes and credible intervals rather than solely on *p*-values.

Given the exploratory purpose of this pilot study, results should be interpreted as tentative signals of association, intended to generate hypotheses for future research rather than confirm hypotheses or predictive relationships.

To ensure clarity and alignment between research questions, the applied statistical tests and the analyzed variables, the following analytical plan was adopted (Table 1).

## 3. Results

### 3.1. Descriptive Statistics

The study sample comprised 20 players, categorized into four position groups: goalkeepers (N = 2), defenders (N = 7), midfielders (N = 5) and forwards (N = 6).

Descriptive statistics (means and standard deviations) for psychological variables—including ACSI-28 total and subscale scores, and RS total and subscale scores—alongside performance indicators, were calculated for all variables as part of the preliminary analysis.

According to the matrix possibilities for data collection from ADT, and considering the analyzed variables, it makes sense to first understand the average number of players’ ball losses throughout the five matches and, mainly, the standard deviation (Table 2).

Regardless of the variation in the players’ number of losses (between 2 and 29 losses) and knowing the risk of not establishing an observation minimum number to include individuals, given the sample size, it was decided not to define any exclusion criteria of this kind.

The expressed number of losses’ variation in Table 2 can be explained by numerous variables, firstly by the players’ differences in playing time over the five matches, but also by the roles that tended to be performed by each one in a match. The effect of these influences is not negligible and, in future studies, for a more effective control of external variables, the methodology must consider it without giving up ecological principles.

Also regarding to more specific indicators of the actions revealed in ADT, it is necessary to take care of the possible influences of external and uncontrolled variables, such as, for example, the field zone in which the ball is lost or the number of teammates behind the ball line now of loss, which probably changes the perception of the need to maintain recovery intentions or not.

The average percentage for maintaining intentions (% Maintained Intention) is 70.47%, with a high standard deviation (SD = 25.40), suggesting significant variability among players. The average percentage for slowing down/canceling intentions (% Slowed Down/Cancelled Intention) is 29.48%, with almost the same standard deviation, further indicating a broad spread of behaviors.

To address the issue of performance standardization across ADT action categories, we conducted a statistical analysis of the relative percentages of five types of ADT actions performed by the players. A repeated measures ANOVA with Greenhouse–Geisser correction (ε = 0.54) revealed a significant main effect, *p* < 0.001, with a large effect size (partial η^2^ = 0.49). The effect size indicates that nearly 49% of the variance in the dependent variable is explained by the differences across action categories. This demonstrates that players do not perform all action categories with equal frequency.

Action categories were predicted to be performed with different frequencies, and this repeated measures ANOVA allows us to confirm this hypothesis.

### 3.2. Inferential Analyses

#### 3.2.1. ADT Behaviors

To assess whether intentions to maintain or slow down/cancel ball recovery actions differed, a Wilcoxon signed-rank test was performed comparing these percentages within players (Table 3). The test revealed a statistically significant difference (*p* = 0.004), with a larger number of players showing higher maintained intentions than slowed/cancelled intentions. This points to the prevalence of maintaining recovery intentions after ball loss.

#### 3.2.2. Psychological Constructs

To examine whether the players’ psychological resilience (Table 4) was below the high-resilience benchmark (147 points), a one-sample *t*-test (one-tailed) was performed.

Results indicated that the sample scored significantly lower than the cutoff (M = 140.45, SD = 12.09), *t*(19) = −2.42, *p* = 0.013. The effect size was of medium magnitude (Hedges’ *g* = −0.52; 90% CI two-tailed: [−0.89, −0.15], equivalent to a 95% one-tailed CI).

This finding suggests that, on average, the players did not reach the level typically associated with high resilience, as defined by the RS benchmark.

No inferential tests were conducted against external cutoffs for RS Factor I (Personal Competence) and RS Factor II (Acceptance of Self and Life), since no validated benchmarks are available for these factors.

For ACSI-28 data (Table 5), total scores ranged from 36 to 80, demonstrating variability in athletic coping skills across players. Subscale analysis revealed high Availability to Learn from Training and Confidence scores, with greater variability in Confrontation with Adversity and Goal Setting, highlighting areas for potential psychological skills intervention.

### 3.3. Group Comparisons by Player Position

To analyze differences in ACSI-28 subscales by player position (goalkeepers, defenders, midfielders, forwards), assumptions for ANOVA were assessed. Levene’s test confirmed homogeneity of variances (all *p* > 0.05). The Shapiro–Wilk test indicated non-normality for some variables (Performance Under Pressure, *p* = 0.03; Availability to Learn from Training, *p* = 0.02). Therefore, given the small sample size per position group and violated normality, non-parametric Kruskal–Wallis tests were conducted (Table 6 and Table 7).

The Kruskal–Wallis tests, Table 6 and Table 7, revealed no statistically significant differences across player positions for any of the psychological subscales (all *p* > 0.05). Effect size values (ε^2^) were also computed to provide a more complete interpretation of the results. For general resilience (Total Score RS), the test statistic was H(3) = 4.77, *p* = 0.19, with a small-to-medium effect size (ε^2^ = 0.09). For personal competence (Factor I), the result was H(3) = 4.56, *p* = 0.21, with a small-to-medium effect size (ε^2^ = 0.08). For acceptance of self and life (Factor II), the result was H(3) = 2.81, *p* = 0.42, with a negligible effect size (ε^2^ = 0). Taken together, these findings suggest broadly similar psychological profiles across player positions in this sample.

Effect size estimates (ε^2^) for ACSI-28 subscales were all negligible-to-small (ranging from 0.00 to 0.08), reinforcing the absence of meaningful positional differences.

Although Kruskal–Wallis tests were conducted, the very small number of players per position—particularly goalkeepers (N = 2)—renders these comparisons non-informative. These results are reported only for transparency and should not be overinterpreted.

Although it could be hypothesized that psychological profiles would vary by player position, Kruskal–Wallis tests showed no significant differences across positions, suggesting broadly similar psychological profiles. While this hypothesis is not supported, effect size calculations indicate small-to-moderate differences, which may provide useful insights for future studies.

### 3.4. Correlation Analyses

Spearman’s rho correlations were calculated to explore relationships between psychological subscales and total scores. Using a threshold of r > 0.80 to denote very strong correlations, the total ACSI-28 score showed very strong associations with subscales of Confrontation with Adversity (r = 0.84), Goal Setting (r = 0.87), Concentration (r = 0.86), and Confidence and Motivation (r = 0.90). These correlations emphasize the interconnectedness of the players’ core psychological competencies.

Additionally, a moderate positive correlation was found between RS total score and ACSI-28 Confrontation with Adversity (r = 0.48, *p* = 0.03), with a stronger association involving RS Factor 1 (self-confidence, determination) and the same ACSI subscale (r = 0.57, *p* = 0.01), linking resilience to coping skills.

### 3.5. Bayesian Analysis

Given the small sample size (N = 20), Bayesian analysis was employed as it provides inferential advantages under limited data conditions [35,36,37]. Unlike frequentist statistics, which relies on asymptotic properties and larger samples to ensure statistical power, Bayesian inference incorporates prior distributions and derives posterior distributions for the parameters of interest. This enhances the interpretability of estimates and allows direct quantification of evidence in favor of competing hypotheses via Bayes Factors (BF).

For the % Less1sec variable, a frequentist ANOVA indicated no statistical significance, F(15,4) = 2.01, *p* = 0.26. Bayesian model comparison similarly yielded BF_10_ = 0.06, indicating strong evidence in favor of the null model. Posterior estimates had wide 95% credible intervals and means close to zero, suggesting no substantial association with psychological predictors.

For the % Maintained Intention variable, the regression model was also non-significant, F(15,4) = 2.55, *p* = 0.19. Bayesian analysis yielded BF_10_ = 0.13, again favoring the null over the predictor model, with posterior parameter estimates remaining small and imprecise.

Despite this overall tendency for Bayes Factors to support the null model, posterior distributions indicated possible positive associations in two specific cases:

- Concentration showed a positive relationship with % Maintained Intention (Mode = 0.44; 95% CI [0.03, 0.72]);
- Goal Setting showed a positive relationship with % Less1sec (Mode = 0.47; 95% CI [0.06, 0.73]).

In both cases, the posterior credible intervals excluded zero, suggesting that these skills might be linked to recovery-related actions. However, because the Bayes Factors favored the null model overall, such findings should be interpreted as exploratory signals rather than robust evidence.

In summary, the Bayesian analysis reinforces that most psychological constructs—including psychological resilience—did not show reliable associations with ADT recovery behaviors. The few exceptions (Concentration and Goal Setting) highlight potential avenues for future research but require confirmation in larger and more diverse samples.

## 4. Discussion

### 4.1. Overview

While the results should be interpreted with caution due to design and statistical limitations that warrant further investigation, the findings help support the original research objectives and suggest the following key assumptions:

Although players did not perform all action categories with equal frequency during ADT, which can be considered expected, there was a clear prevalence of maintaining recovery intentions following ball loss. It was also possible to note substantial variability in psychological measures, with most RS scores generally below the threshold for high resilience (147) with a medium effect size (g = −0.52), which may indicate the need for individualized interventions and suboptimal levels of psychological resilience.

No statistically significant differences were found in psychological competencies/skills (as measured by RS and ACSI-28) across different playing positions at this age.

A moderate positive correlation was identified between the RS total score and the ACSI-28 “Confrontation with Adversity” subscale. This association was more pronounced between RS Factor 1 and the same ACSI-28 subscale.

Although some psychological variables showed preliminary associations with individual behaviors during ADT, it would be premature to assert that these competencies/skills are the sole or primary predictors of success in such situations. The study design prevents establishing whether these psychological characteristics actively influence behavior, are outcomes of prior experiences, or reflect other contextual mediators (such as tactical variables, for instance).

Goal Setting (defined as the ability to set short-term goals, pursue performance objectives and mentally prepare for competition), as well as Concentration (the capacity to focus on tasks even when adverse or unexpected situations occur), showed exploratory signals of association with successful ADT individual behaviors, but the absence of statistical significance and the small effect sizes caution against overgeneralization.

### 4.2. Interpretation of Findings

#### 4.2.1. Research Question 1

Despite all the constraints, the findings highlight several patterns. The wide inter-individual variability in ADT behaviors (the large effect size indicates that differences across action categories account for nearly half of the variance, showing that players do not perform all actions with equal frequency), as well as, in general, psychological resilience and athletic coping skills (coupled with RS scores below the threshold for high resilience (147)) suggests that sporting performance and psychological resources in this population are highly heterogeneous, even for a small and homogeneous sample. This underscores the importance of individualized psychological profiling, enabling the coherent integration of diverse theoretical frameworks and assessment or teaching–learning approaches [19,20,21,22,23,24,25,26,27,28,29,30,31,32,33,34,35,36,37,38], while avoiding standardized, non-contextualized interventions that disregard ecological principles [39].

This evidence corroborates that tailored psychological support in adolescence should be necessary to enhance both sport performance and mental health outcomes [8,9,10,11,12,13,14,15,16,17,18,19,20,21,22].

A key consideration for an accurate interpretation of our findings is the documented challenges associated with using the ACSI-28 and the RS in youth populations. Despite their validation for assessing psychological competencies, their use with younger individuals can lead to several confounding factors. These include the potential for item misinterpretation stemming from a lack of life experience, developmental factors within the psychological domain, and well-known self-report biases, such as social desirability [14,15,16,17,18,19,20,21,22,23,24,25,26,27,28,29,30,31,32]. As such, extreme caution is warranted when interpreting the current results. This observation also underscores the critical need for continued research to establish the psychometric validity of these instruments in this specific population.

#### 4.2.2. Research Question 2

No significant differences in the assessed psychological constructs emerged across playing positions, consistent with prior research in similar age groups [40,41]. While positional demands might intuitively shape psychological traits, these findings suggest such effects may be minimal during the analyzed pre-specialization stage or overshadowed by individual differences.

Conversely, some research in older youth or senior players indicates that positional demands can shape psychological characteristics—for example, goalkeepers often report higher levels of emotional regulation due to the unique stressors of their role [41].

These findings imply that psychological resilience development should be considered a universal priority in team contexts, rather than a position-dependent attribute. Nonetheless, the moderate effect sizes point to potentially meaningful variation that may become evident in larger cohorts. Future research should therefore address this question using adequately powered designs to determine whether subtle positional demands influence resilience profiles.

The lack of positional variation in the present study may also reflect developmental factors: tactical responsibilities and specialized roles could still not be well defined at this age, meaning that individual differences outweigh position-specific influences. This reinforces the idea that psychological interventions in early adolescence should be player-centered rather than role-specific.

#### 4.2.3. Research Question 3

The moderate positive correlation between RS Factor 1 (determination, perseverance, and related traits) and the ACSI-28 “Confrontation with Adversity” subscale suggests an alignment between individuals’ general resilience and their sport-specific adversity coping skills. However, the modest strength of this relationship reinforces the notion that other factors beyond psychological resilience likely influence players’ responses to stressors in sports environments and, consequently, that ADT individual behaviors in football might be shaped by a constellation of interacting factors, not psychological traits alone [11].

Similar patterns have been observed in adolescent and young adult samples, where measures of resilience correlate moderately with coping subscales, but not strongly enough to be considered redundant [8,9,10]. The moderate strength of the correlations found here underscores that while psychological resilience and some athletic coping skills are related, they possibly capture distinct psychological dimensions. Psychological resilience is a broader construct, whereas athletic coping skills, as assessed by the ACSI-28, are specifically anchored in sport-related challenges. This finding adds nuance to existing literature by showing that even within a homogeneous sample of U14 players, different psychological constructs provide complementary information.

#### 4.2.4. Research Question 4

As was highlighted before, the behavioral data indicated that players showed a significant tendency to sustain ball-recovery intentions following possession loss. This finding resonates with research that underlines ADT as particularly revealing of motivation, persistence and psychological readiness [11,12,13,14,15,16,17,18,19].

To perceive the possible relation of psychological resilience and athletic coping skills with players’ behaviors during ADT, a Bayesian analysis was applied (Bayesian inference and Bayes Factor). The Bayesian analyses suggested possible positive associations for ‘Concentration’ and ‘Goal Setting’, as their posterior distributions indicated credible intervals excluding zero. However, Bayes Factors still favored the null model overall (Bayes Factors < 0.2), meaning that these results should be regarded as preliminary signals rather than robust evidence.

By contrast, most psychological constructs, including psychological resilience, showed no reliable evidence of correlation with recovery-related actions. In these cases, Bayes Factors consistently supported the null hypothesis or yielded inconclusive evidence, suggesting limited direct associations of these traits with recovery behavior.

This initial finding provides a slight indication of the potential association that certain psychological competencies/skills may have with players’ behaviors in perceived high-stress situations during football matches [11]. Nevertheless, the absence of strong or consistent associations between these constructs and specific ADT actions also supports the argument that behavior in complex match situations emerges from the interaction of multiple constraints (technical, tactical, psychological, and environmental) rather than from psychological traits in isolation. This aligns with the growing literature calling for complexity-informed and ecological approaches to performance analysis in football [18,19,20].

#### 4.2.5. Research Question 5

Within the applied analysis, ‘Goal Setting’ and ‘Concentration’ emerged as the only constructs showing preliminary signals of a possible association. These signals suggest that players with stronger goal orientation and mental preparation may be more consistent in initiating rapid recovery actions at ADT, although this requires confirmation in larger samples. On the other hand, players who demonstrate better attentional focus—even when facing competitive stressors—might have a higher tendency to sustain their recovery actions during ADT, but the evidence remains inconclusive.

This finding aligns with studies emphasizing the role of goal setting and self-regulation in supporting effective match behaviors [2,3,4,5]. Nevertheless, since Bayes Factors favored the null model (Bayes Factors < 0.2), these results must be regarded as exploratory indications rather than reliable associations, underscoring the multifactorial nature of ADT behaviors—psychological skills likely interact with tactical understanding, technical ability, physical readiness and situational constraints.

From a practical standpoint, this suggests that psychological profiling should be integrated in a broader diagnostic approach, rather than used in isolation, to predict and support performance in football match transitions.

#### 4.2.6. Synthesis

By addressing the five research questions explicitly, the present study demonstrates that psychological constructs such as psychological resilience and athletic coping skills could provide valuable but incomplete insights into performance during football ADT.

The findings are consistent with the international literature highlighting the interplay of different performance factors and contextual variables in shaping youth players’ behavior [18].

Importantly, the integration of psychological assessment with match analysis of individual behaviors emerges as a promising methodological approach [19], advancing both the scientific understanding of youth football performance and the design of applied interventions aimed at optimizing development and supporting mental health.

However, in an exploratory analysis like this, results must be clearly identified as speculative and warrant further investigation with a developed hypothesis revealed during the analysis, a key concept that is often forgotten [37].

It was not possible to conclude that higher psychological resilience and athletic coping skills alone would lead players to display more consistent defensive intentions and recovery-oriented behaviors during ADT. Nevertheless, the importance of further investigating these variables was reinforced, particularly regarding their interaction with other performance factors and dimensions (individual, task and environment).

### 4.3. Theoretical Implications

This research advances the integration of psychological constructs into match analysis—a methodological gap noted in performance science [42,43]. The results align with contemporary perspectives viewing psychological resilience as a dynamic, context-dependent process shaped by individual traits, social support and environmental demands [4,5,6,7,8,9,10,11]. They also support the argument for multifactorial models of sport performance, where psychological variables come together, but do not supersede, tactical, technical and physiological dimensions.

By linking psychological assessment with behavioral indicators, this study contributes to the development of diagnostic and intervention frameworks capable of informing both performance optimization and players’ holistic development [44].

This is particularly relevant because sports performance is not limited to physical issues: practitioners’ mental health also holds enormous importance [45].

Such approaches transcend isolated questionnaire-based evaluations, fostering integration between the scientific domains that collectively inform the study of sport.

### 4.4. Practical Applications

It is well established that aggregated data alone may be insufficient to enhance individual performance, as performance indicators must be individualized and contextualized, and the predictive capacity of human behavior remains limited unless data analysts and sport psychology researchers collaborate closely [19,20,21,22,23,24,25,26,27,28,29,30,31,32,33,34,35,36,37,38,39,40,41,42,43]. Accordingly, this study can be viewed as an effort to operationalize these principles within a novel methodological framework.

Although preliminary, these findings underscore the eventual potential of integrating psychological assessment with behavioral analysis in youth football, thereby contributing to a holistic understanding of this phenomenon. Future research could test whether individualized interventions targeting coping skills and resilience-related competencies, when integrated into training programs, might support both performance and mental health outcomes [45,46]. Importantly, such programs should be embedded within football-specific contexts to ensure ecological validity and avoid the pitfalls of generic and decontextualized psychological training.

The preliminary associations identified for “Goal Setting” and “Concentration” may suggest that interventions on these domains should be further investigated to enhance the knowledge about the practical effects of strengthening these specific skills.

The design of goal-setting programs may constitute a relevant hypothesis to be tested in an experimental framework. Coaches and sport psychologists might assist players in defining short-term, specific and measurable behavioral goals during training (e.g., “initiate actions to regain possession within one touch after losing the ball”), and progressively integrate these goals into match-like scenarios, evaluating the possible effects of this intervention on performance.

Similarly, with respect to “Concentration”, training tasks could be structured to promote this capacity and subsequently evaluate their impact on match performance. For instance, exercises such as attention switching drills—requiring players to rapidly alternate between offensive and defensive tasks in response to diverse stimuli—may constitute the core of an intervention protocol to test the possible influences on players’ ability to maintain focus under pressure and to recover effectively after mistakes.

These applications should be incorporated into regular practice environments without disrupting tactical, technical or physical training. For instance, small-sided games can be adapted to reward persistence in recovery actions, thereby reinforcing psychological dimensions simultaneously with other performance factors. Likewise, integrating brief reflective discussions after matches based on specific and analyzed behavioral indicators—where players could observe how they responded to moments of pressure like ADT—may strengthen the interpretation of psychological resilience and coping skills in ecologically valid contexts.

Such examples show how psychological profiling should not be an isolated diagnostic tool but rather integrated into broader training routines, combining technical, tactical and physical preparation. In this way, sport psychology can move from abstract and non-contextualized assessments to actionable strategies that support both performance optimization and youth players’ holistic development.

Participation in sport inevitably involves both benefits and challenges, even when employed to foster psychological competencies and promote mental health among youth. This study aims to contribute to maximizing these benefits by advancing the understanding of how specific psychological capacities relate to their behavioral expressions. Such knowledge may enhance the effectiveness of interventions, adopting an ecological approach that spans the full process of diagnosis, intervention, and evaluation [46].

### 4.5. Limitations

#### 4.5.1. Sample and Statistical Limitations

The present study represents an exploratory attempt to integrate psychological profiling with a specific match analysis in youth football. However, several limitations warrant careful consideration.

The modest, homogeneous sample (N = 20), drawn from a single U14 male team, naturally constrains generalizability and statistical power, particularly for some positional subgroups (e.g., goalkeepers). The fact that some subgroups consisted of a relatively small number of participants may have influenced the obtained results, potentially preventing the identification of differences that could have been unveiled with a larger or different sample. However, the available sample represents a complete team and is comparable to similar studies in youth sports contexts [35,36].

Another important limitation of this study concerns the number of statistical tests performed across several psychological and behavioral variables. Although this breadth is consistent with the exploratory scope of the work, it increases the risk of Type I error and the likelihood of identifying false positives. The reliance on Bayes Factors mitigates this risk to some extent, yet none of the associations would survive a conservative correction for multiple comparisons. Therefore, all reported findings should be interpreted as preliminary signals that require replication in larger and more robust datasets before any firm conclusions can be drawn.

#### 4.5.2. ACSI-28 and RS Limitations

Although the ACSI-28 and the RS are validated tools, their use with early adolescents presents some challenges. Misinterpretation of items, developmental differences, and potential self-report biases (e.g., social desirability) may have affected results and have been reported in prior studies [14,15,16,17,18,19,20,21,22,23,24,25,26,27,28,29,30,31,32,33,34,35,36,37,38,39,40,41,42,43,44,45,46,47].

These factors underscore the need for cautious interpretation of the achieved results and enhance the need for methodological refinement before firm conclusions can be drawn about these age group samples, considering the explored psychological constructs. Mixed-method approaches, combining self-report with interviews or behavioral validation, may be a hypothesis to consider in future studies.

#### 4.5.3. Potential Coding Bias and Ecological Constraints

In addition to the constraints already discussed, several further limitations must be acknowledged.

Although the observational grid was validated and applied consistently by the two observers, potential coding bias cannot be excluded.

Contextual factors such as variability in playing time, ball-loss location, teammate/opponent density at ball-loss moment, match score, opposition quality or tactical instructions were not controlled in this study, and these may have influenced players’ behaviors during ADT, introducing variability that cannot be attributed solely to psychological constructs.

These variables may reflect differences in players’ exposure to possession losses (e.g., some experiencing considerably fewer recovery opportunities) and introduce additional variability, thereby limiting comparability and results interpretations.

Together, these factors reinforce the exploratory character of the study and the need for replication with larger, diverse samples and more controlled observational designs.

### 4.6. Future Directions

To build on these insights and overcome current limitations, future research should:

Expand and diversify samples, encompassing different age groups, competitive levels and genders to enhance generalizability;

Adopt longitudinal and experimental designs, enabling the examination of causal relationships between psychological traits and behavioral outcomes;

Integrate contextual match variables (e.g., pitch zones, team structures, opposition quality) to refine behavioral analyses during ADT;

Use mixed methods approaches, combining quantitative behavioral and psychological metrics with qualitative insights (e.g., interviews, observations) to capture players’ lived experiences of stress and adaptation;

Test intervention frameworks, embedding psychological resilience and athletic coping training within ecologically valid football programs, to evaluate their impact on both performance and youth mental health.

On the other hand, building on the findings from this study, future research could transition from exploratory model-building to more theoretically driven frameworks, particularly with larger datasets. While our analysis utilized exploratory methods to identify possible relationships, an expanded investigation with a larger sample would benefit from a hierarchical or structural regression approach. This would not only enhance the robustness and interpretability of the results but also allow for the explicit testing of theoretical pathways. Such a shift from data-driven discovery to theory-driven confirmation would provide a more rigorous and coherent understanding of the underlying mechanisms, offering stronger evidence for future interventions and policies.

In summary, while this pilot study offers only preliminary signals, future research should employ longitudinal and experimental designs with larger and more diverse samples to test whether these associations hold. Incorporating contextual match variables and mixed-method approaches will be crucial to move from exploratory findings towards confirmatory evidence that can more reliably inform practice in youth football.

By addressing these priorities, future studies can refine the proposed holistic methodological framework, strengthen the evidence base and advance interdisciplinary performance models that more accurately capture the psychological underpinnings of youth football.

## 5. Conclusions

This exploratory study, adopting an ecological approach, sought to integrate psychological dimensions (specifically psychological resilience and athletic coping skills) into match analysis of youth football players during ADT. Although the findings offer preliminary insights into possible associations—particularly for “Goal Setting” and “Concentration”—the overall evidence remains inconclusive and their interpretation must be approached with caution given several methodological constraints: the reduced sample, the limited number of analyzed matches and the exploratory correlational design, which precludes causal inferences.

Additionally, unmeasured factors such as tactical demands, technical execution and physiological state may have significantly influenced the observed behaviors, potentially confounding the relationships identified.

It is also important to note that, given the number of comparisons conducted, the results should be viewed as preliminary and potentially inflated by Type I error. Replication with larger samples and more stringent analytic controls will be essential to determine whether the observed associations are reliable.

In summary, although speculative, this study provides an initial step towards advancing research on the feasibility of incorporating equally all the performance factors into football performance diagnostics. Nevertheless, the current evidence base is insufficient to support definitive conclusions regarding the explored relationships or even their practical applications.

Notwithstanding, this work suggests that some psychological constructs may be associated with individual player behaviors during competitive scenarios. The evidence was largely inconclusive, with only preliminary indications for “Goal Setting” and “Concentration”, but it represents a step towards reinforcing the need for research about these issues.

Overall, the present results should be interpreted as hypothesis-generating rather than hypothesis-confirming, offering initial indications of possible associations that require replication in larger and more controlled studies.

Future research should employ longitudinal and experimental designs, larger and more diverse cohorts, and integrated models capturing tactical, technical, physiological and psychological domains. Only through such multifactorial and methodologically rigorous approaches can the true role of psychological resilience and athletic coping skills in shaping youth football performance (and their implications for mental health promotion) be accurately determined.

## Figures and Tables

**Table 1:** Overview of statistical analyses conducted in relation to the research questions.

Research Question	Statistical Analysis	Independent Variables	Dependent Variables
Are there systematic differences between types of performed ADT actions? (Characterize players’ individual behaviors during ADT)	Repeated Measures ANOVA (Greenhouse–Geisser)	ADT action category (% Not Initiated; % Less1sec; % More1sec; % Maintained Intention; % Slowed Down/Canceled Intention)	Relative frequency of each action type
Do players tend to maintain recovery intentions after possession loss? (Characterize players’ individual behaviors during ADT)	Wilcoxon signed-rank test	% Maintained Intention vs. % Slowed/Cancelled Intention	Within-player paired percentages (recovery effort indicators)
Do players’ resilience levels differ from the high-resilience benchmark (147)? (Characterize players’ psychological profiles)	One-sample *t*-test (one-tailed)	Benchmark value (147)	RS total score
Are there positional differences in ACSI-28 and RS scores? (Examine positional differences)	Kruskal–Wallis	Player position (categorical: GK, DEF, MID, FWD)	ACSI-28 and RS scores
Are coping skills associated with resilience? (Explore associations between ACSI-28 coping dimensions and RS scores)	Spearman’s rank correlations	ACSI-28 total score and subscales scores RS total score and two-factor scores	
Are psychological constructs associated with behavioral indicators during ADT? (Investigate the relationships between resilience, athletic coping skills and players’ behaviors during ADT)	Bayesian inference through Bayes Factors	ACSI-28 total score and subscales scores RS total score and two-factor scores % Less1sec % Maintained Intention	
Which psychological dimensions best predict ADT performance? (Identify psychological dimensions most strongly associated with behavioral success in ADT)	Bayesian inference through Bayes Factors	ACSI-28 total score and subscales scores RS total score and two-factor scores % Less1sec % Maintained Intention	

**Table 2:** Actions’ percentages at ADT revealed by players in relation to the total number of losses.

	Total Losses	% Not Initiated	% Less1sec	% More1sec	% Maintained Intention	% Slowed Down/Canceled Intention
Average	9	12.30	61.86	25.84	70.47	29.29
Standard Deviation	6.70	15.72	25.56	23.73	25.40	25.48
Maximum	29	55.56	100.00	100.00	100.00	77.78
Minimum	2	0.00	0.00	0.00	22.22	0.00

**Table 3:** Wilcoxon signed-rank test results comparing percentages of Slow Down/Cancelled Intentions with maintained intentions at ADT.

Ranks	N	Mean Rank
Negative Ranks	16	11.38
Positive Ranks	4	7.00
	% Maintained Intention—% Slowed Down/Cancelled Intention	
Significance Level	0.004	

**Table 4:** Total score and two-factor scores achieved on RS.

	Total Score RS	Factor I	Factor II
Average	140.45	99.45	40.95
Standard Deviation	12.09	8.85	6.12
Maximum	165	112	55
Minimum	116	81	32

**Table 5:** Total score and subscales’ scores achieved on ACSI-28.

Player	Total Score ACSI-28	Confr. with Adversity	Max. Perf. Under Pressure	Goal Set.	Concent.	Abs. of Worries	Confid. and Motiv. to Achieve	Avail. to Learn from Train.
Average	57.60	8.20	8.95	7.60	8.10	5.50	9.40	9.85
Standard Deviation	12.38	2.61	2.48	3.17	1.89	2.46	1.88	2.13
Maximum	80	12	12	12	12	12	12	12
Minimum	36	2	4	1	5	2	6	5

**Table 6:** Kruskal–Wallis test results for RS considering players’ position in the field.

	Total Score RS	Factor I	Factor II
Kruskal–Wallis H	4.77	4.56	2.81
df	3	3	3
Asymp. Sig.	0.19	0.21	0.42

**Table 7:** Kruskal–Wallis test results for ACSI-28 considering players’ position in the field.

	TOTALACSI28	ConfAdvs.ACSI28	Max. Perf. Under Pressure	Goal Set.ACSI28	Concent.ACSI28	A.Worries.ACSI28	Conf.Motv.ACSI28	Avail. To Learn From Train.
Kruskal–Wallis H	0.21	1.98	2.88	0.35	1.63	2.23	1.02	1.13
df	3	3	3	3	3	3	3	3
Asymp. Sig.	0.98	0.58	0.41	0.95	0.65	0.53	0.80	0.77

## Data Availability

The data that support the findings of this study are available on reasonable request from the corresponding author, F.P. The data are not publicly available due to privacy and ethical restrictions.

## Abbreviations

The following abbreviations are used in this manuscript:

ADT	Attack–Defense Transition Moments
ACSI-28	Athletic Coping Skills Inventory
RS	Resilience Scale
MLR	Multiple Linear Regression analysis

## Appendix A

Firstly, to outline possible interpretations that allows to detect match transition moments, it was defined that “In duel/dispute situations, possession is considered to change (with DAT/ADT) when the result benefits the team that initially did not have the ball (2” following the dispute as reference time and 1 successful pass after the dispute as reference action). Situations in which the ball goes out are excluded.”

In this way, the aim was to define, through easily observable indicators, a pattern that makes it possible to distinguish with relative simplicity the moments that may or may not be included in the analysis, despite the possible discussion about the used criteria.

This results in an analysis matrix with two sets of actions perfectly identified (Table A1).

**Table A1:** Set of actions in the analysis matrix related to the DAT and to the ADT.

DAT						ADT		
Action Beginning	Ball Conduction		Pass		Shot	Action Beginning	Result	Intention
	Direction	Result	Direction	Result	Result			

To begin scalping the data related to ADT, and based on how the “Time” variable can be unfolded into actions in these match moments, the player’s “Action Beginning” was identified as the first analysis reference (Table A2), categorizing 3 types of actions:

- “No action initiated”;
- “Less than 1” after the last touch”;
- “More than 1” after the last touch”.

These indicators, structured around the embodiment of the “Time” variable (knowing that “Space” = “Velocity” × “Time”), make it possible to differentiate players’ tendencies at this critical match moment, in which the temporal dimension associated with its definition underlines the urgency of speed in players’ actions.

**Table A2:** Division of the “Action Beginning” indicator in the analysis of the ADT.

ADT
Action Beginning:
No action initiated
Less than 1” after the last touch More than 1” after the last touch

Although “Velocity”, naturally associated with players’ “Action Beginning”, is considered a critical factor in this match moment, itself might not offer enough information to better characterize the match and the players (even so, it could be tested in an isolated way, trying to perceive its potential influence), making it necessary to cross indicators related to success/failure of the diverse possible sequential actions.

So, the categorization of the predominantly used actions (related to individual technical-tactical actions), their results, or even their revealed intention, is very important. In Table A3, it is possible to observe the ADT “Result” and “Intention” categorization.

**Table A3:** Division of action indicators in the analysis of the ADT.

ADT	
Result	Intention
Did not intercept	Maintained intention to recover after 3 touches from the opponent
Intercepted/recovered the ball (gained possession) within 3 touches of the opponent	Slowed down or canceled the intention to recover after 3 touches from the opponent
Intercepted/recovered the ball (gained possession) after 3 touches from the opponent	
Foul	

Considering that recovering ball possession becomes ADT priority goal collectively and individually, it was decided to define as an indicator of the actions carried out following this moment the “Result”, outlining 4 possibilities:

- “Did not intercept”, when the player who loses ball possession is unable to intercept or recover it;
- “Intercepted/recovered the ball (gained possession) within 3 touches of the opponent”, when the player who loses ball possession intercepts or recovers it before the opposing team touches the ball 3 times;
- “Intercepted/recovered the ball (gained possession) after 3 touches from the opponent”, when the player who loses ball possession intercepts or recovers it after the opposing team touches the ball 3 times;
- “Foul” is when the player who loses ball possession makes a foul.

In this way, beyond the success or failure of the actions taken following the transition moments, it could be possible to obtain some information (using limits like the number of touches in the indicators’ definition) about the time variation in which each player tends to act.

Furthermore, and extending the analysis in time (beyond the opponent’s 3 touches) to better understand the players’ attitude towards all the generated interactions following ADT, it was necessary to collect data about their “Intention” when time passes by, defining two options:

- “Maintained intention to recover after 3 touches from the opponent”, when the player who loses ball possession remains committed to carrying out his defensive tasks;
- “Slowed down or cancelled the intention to recover after 3 touches from the opponent”, when the player who loses ball possession stops carrying out his defensive tasks or slows down the speed with which he tries to accomplish it.
